# Extended Phase-Space Methods for Enhanced Sampling in Molecular Simulations: A Review

**DOI:** 10.3389/fbioe.2015.00125

**Published:** 2015-09-03

**Authors:** Hiroshi Fujisaki, Kei Moritsugu, Yasuhiro Matsunaga, Tetsuya Morishita, Luca Maragliano

**Affiliations:** ^1^Department of Physics, Nippon Medical School, Tokyo, Japan; ^2^Graduate School of Medical Life Science, Yokohama City University, Yokohama, Japan; ^3^Advanced Institute for Computational Science, Kobe, Japan; ^4^National Institute of Advanced Industrial Science and Technology (AIST), Tsukuba, Japan; ^5^Center for Synaptic Neuroscience, Istituto Italiano di Tecnologia, Genova, Italy

**Keywords:** molecular dynamics, rare events, free energy, biomolecular interactions, conformational transitions, protein–ligand binding

## Abstract

Molecular Dynamics simulations are a powerful approach to study biomolecular conformational changes or protein–ligand, protein–protein, and protein–DNA/RNA interactions. Straightforward applications, however, are often hampered by incomplete sampling, since in a typical simulated trajectory the system will spend most of its time trapped by high energy barriers in restricted regions of the configuration space. Over the years, several techniques have been designed to overcome this problem and enhance space sampling. Here, we review a class of methods that rely on the idea of extending the set of dynamical variables of the system by adding extra ones associated to functions describing the process under study. In particular, we illustrate the Temperature Accelerated Molecular Dynamics (TAMD), Logarithmic Mean Force Dynamics (LogMFD), and Multiscale Enhanced Sampling (MSES) algorithms. We also discuss combinations with techniques for searching reaction paths. We show the advantages presented by this approach and how it allows to quickly sample important regions of the free-energy landscape via automatic exploration.

## 1 Introduction

Molecular dynamics (MD) simulations have become a fundamental tool to study biological systems at atomic scale (Perilla et al., 2015). MD allows to simulate the motion of each single atom of complex biomolecules in accurately modeled environments, thus providing information about molecular mechanisms at a scale which is still impossible to access experimentally. Such information can in turn be of extreme value to design new, more insight-driven experiments. A major difficulty in MD is, however, the well known time-scale issue: most of the phenomena of (biophysical) interest occur on times that are still unaccessible by standard simulations. Few remarkable exceptions exist (Freddolino et al., 2010; Lindorff-Larsen et al., 2011), although even in these cases it remains challenging to accrue enough statistics for a comprehensive understanding of the process.

The problem originates from the presence of dynamical hindrances of energetic or entropic nature, which confine the system in specific regions of phase space. Transitions among those metastable states, while being *rare events* on the simulation timescale, are often a mandatory requirement for biological function. This is the case, for example, of a receptor changing conformation to accommodate a ligand, or of two associating proteins in solvent forming the encounter complex.

A thermodynamic quantity of pivotal importance in MD simulations of rare events is the free energy, or potential of mean force (Roux, 1995), since it provides information on the metastable states and the transitions between them. In particular, via the free energy it is possible to obtain the relative probability of different molecular conformations, or the rate at which dynamical transitions occur between them. From its statistical mechanics definition, the free energy is related to the probability density of a set of collective variables (CVs), which are typically used to study a reactive event. These are functions of the Cartesian variables of the original system, such as distances and angles between atoms, or more complicated functions. Unfortunately, due to the sampling issue described above, free-energy calculations are quite demanding. Many numerical methods have been developed in order to accelerate, or enhance, sampling in MD simulations and thus facilitate free-energy calculations. Notable examples include umbrella sampling (Torrie and Valleau, 1974), metadynamics (Laio and Parrinello, 2002), accelerated molecular dynamics (Hamelberg et al., 2004), adaptive biasing force (Darve and Pohorille, 2001), or replica exchange methods (Fukunishi et al., 2002). For a recent review, see Bernardi et al. (2015).

In this review, we illustrate a number of methods that were developed in recent years to reconstruct free-energy surfaces and transition pathways between regions over them. The distinctive feature of these methods is that they are all based on the introduction of an extended system where a set of extra variables is evolved dynamically together with the variables of the physical system. Extending the thermodynamic ensemble of the simulated system is a compelling strategy in computational chemistry/physics, and it has been exploited for example to control temperature and pressure (Nosé, 1984; Hoover, 1985), calculate chemical free energies (Kong and Brooks, 1996; Bitetti-Putzer et al., 2003), or enhance sampling by introducing *generalized ensembles* made of multiple replicas of the full physical system (Mitsutake et al., 2001). In the present context, the added variables are typically associated to the CVs, and evolved together with the physical variables in either a sequential or a concurrent setting. This strategy can be considered within the framework of multiscale computational approaches (E et al., 2007a), and presents several advantages, which are discussed in this review. In the next sections, we divide the discussed methods in two classes. First, we examine techniques designed to explore and reconstruct free-energy surfaces, while afterwards we illustrate methods devised to determine optimal reaction pathways between metastable states.

## 2 Methods to Explore and Reconstruct the Free-Energy Landscape

Let us consider a system whose configurational state is specified by $\text{x}\in R^{N}$, and suppose we are interested in the statistical behavior of a set of collective variables ***θ***(***x***) = (*θ*_1_(***x***), … , *θ_n_*(***x***)). If ***z*** = (*z*_1_, … , *z_n_*) is a particular realization of these variables, the free energy *F*(***z***) of the system is defined as

(1) $$F\left(\text{z}\right)=-\beta^{-1}\text{ ln}\;\left(Z^{-1}\int_{R^{N}}\;e^{-\beta V\left(\text{x}\right)}\prod_{\alpha =1}^{n}\;\delta \left(\theta_{\alpha }\left(\text{x}\right)-z_{\alpha }\right)d\text{x}\right)\;,$$

where $Z=\int_{R^{N}}\;e^{-\beta V\left(\text{x}\right)}d\text{x}$, *V* (***x***) is the potential energy of the system and *β* = 1/*k_B_T*, where *k_B_* is the Boltzmann constant and *T* the temperature. Note that *F*(***z***) depends on a specific temperature *β*^−1^, determined, for example, based on experimental information on the process to be studied. The definition equation (1) implies that *e^−βF^*^(^*^***z***^*^)^ is the probability density function of the variables ***z***.

A natural way to reconstruct *F*(***z***) would be to consider an artificial dynamics where the variables ***z*** are evolved on the free-energy landscape *F*(***z***) defined at *β*^−1^ using an artificial temperature ${\bar{\beta }}^{-1}$ higher than the physical one. This can be realized for example using the dynamics

(2) $$\mu_{\alpha }{\ddot{z}}_{\alpha }=-\frac{\mathrm{\partial F}\left(\text{z}\right)}{\partial z_{\alpha }}+\text{thermostat at}\;{\bar{\beta }}^{-1}\;,$$

where *μ_α_* is the artificial mass of the fictitious particle α, and $\bar{\beta }=1∕k_{B}\bar{T}$ is the inverse of an artificial temperature $\bar{T}$, higher than physical temperature *T* (more details will be given below). The choice of the thermostat in equation (2) has no effect on the argument discussed here, and one could use Langevin, or Nosé-Hoover (Nosé, 1984; Hoover, 1985) or related techniques (Martyna et al., 1992; Liu and Tuckerman, 2000; Morishita, 2010). Since the equilibrium density of the dynamics equation (2) is proportional to $e^{-\bar{\beta }F\left(\text{z}\right)}$, by setting ${\bar{\beta }}^{-1}>\beta^{-1}$ the ***z***(*t*) solution of equation (2) will meander through *F*(***z***) being able to cross energy barriers higher than *β*^−1^, thus visiting also regions unaccessible to the physical system with thermal energy *β*^−1^.

Such approach is indeed possible, at least in principle, even if the full *F*(***z***) is not known, since the quantity −∂*F*(***z***)/∂*z_α_* (called the *mean force*) can be calculated locally at any point ***z***, via constrained simulations on ***θ***(***x***) = ***z*** using the *blue moon* ensemble method (Carter et al., 1989; Ciccotti et al., 2005). In fact, this idea is at the basis of the methods that we will review in the next sections.

### 2.1 Temperature accelerated molecular dynamics (TAMD)

#### 2.1.1 Overview of the Method

As anticipated at the end of the previous section, integrating equation (2) can be an efficient way to explore the unknown free-energy surface *F*(***z***), since the mean force −▽*F* at a point ***z*** can be estimated via a conditional expectation on ***θ***(***x***) = ***z*** (Carter et al., 1989; Ciccotti et al., 2005), even when *F*(***z***) is unknown. This approach, however, has two difficulties: first, after each integration step providing the updated ***z*** values, the configuration of the physical system may be far from satisfying the new constraints [i.e., ***θ***(***x***) will be different from ***z***], which may lead to re-initialization problems; second, it requires to determine the length of the time-averaging window to compute ▽*F*, which is an extra parameter to adjust.

To overcome these difficulties, in TAMD (Maragliano and Vanden-Eijnden, 2006), it is proposed to simulate effectively equation (2) by using the following extended system of equations

(3) $$\left\{\begin{array}{c} m_{i}{\ddot{x}}_{i}=-\frac{\mathrm{\partial V}\left(\text{x}\right)}{\partial x_{i}}\;-\;\kappa \;\sum_{\alpha =1}^{n}\;\;\;\left(\theta_{\alpha }\left(\text{x}\right)\;-z_{\alpha }\right)\frac{\partial \theta_{\alpha }\left(\text{x}\right)}{\partial x_{i}}\;+\;\text{thermostat}\;\text{at}\;\beta^{-1},\;\\ \mu_{\alpha }{\ddot{z}}_{\alpha }=\kappa \left(\theta_{\alpha }\left(\text{x}\right)-z_{\alpha }\right)+\text{thermostat}\;\text{at}\;{\bar{\beta }}^{-1},\; \end{array}\right.$$

where κ is a parameter whose role will be specified later, and *m_i_* is the mass of the atom *i*. We note that the specific dynamics of the ***x*** and ***z*** variables in equation (3) is not important. In the original formulation, TAMD was presented using overdamped Langevin in equations (3) and (2), while several further applications have shown the effectiveness of other dynamical schemes, such as second order Langevin (Monteferrante et al., 2008), or Nosé-Hoover (Trigila, 2007). For simplicity, we use here second order dynamics for both ***x*** and ***z*** leaving the thermostatting technique unspecified.

The system (3) describes the evolution of the extended system (***x***, ***z***) under the effect of the potential energy

(4) $$U_{\kappa }\left(\text{x},\text{z}\right)=V\left(\text{x}\right)+\frac{1}{2}\kappa \sum_{\alpha =1}^{n}\;{\left(z_{\alpha }-\theta_{\alpha }\left(\text{x}\right)\right)}^{2}.$$

In the TAMD paper (Maragliano and Vanden-Eijnden, 2006), it was shown why a system like equation (3) can be used to sample the free energy *F*(***z***). Here, we summarize the results and refer to the original paper the reader interested in the details. The key argument is that if in equation (3) the artificial masses *μ_α_* are chosen such that *μ_α_ > > m_i_*, then the variables ***z***(*t*) evolve much more slowly than the ***x***(*t*), feeling only their average effect. As a consequence, the variables ***z***(*t*) evolve according to an effective equation which is obtained by averaging the ***z***(*t*) equations (3) with respect to the probability density function for ***x***(*t*) from equation (3) at ***z***(*t*) = ***z*** fixed. More in detail, if we introduce the quantity

(5) $$F_{\kappa }\left(\text{z}\right)=-\beta^{-1}\text{ ln}\left(Z_{\kappa }^{-1}\int_{R^{N}}\;e^{-\beta U_{\kappa }\left(\text{x},\text{z}\right)}d\text{x}\right),$$

with $Z_{\kappa }=\int_{R^{N}\times R^{n}}\;e^{-\beta U_{\kappa }\left(\text{x},\text{z}\right)}d\text{x}d\text{z}$, if *μ_α_* > > *m_i_* the variables ***z***(*t*) solution of equation (3) satisfy approximately the effective equation

(6) $$\mu_{\alpha }{\ddot{z}}_{\alpha }=-\frac{\partial F_{\kappa }\left(\text{z}\right)}{\partial z_{\alpha }}+\text{thermostat}\;\text{at}\;{\bar{\beta }}^{-1},$$

where

(7) $$-\frac{\partial F_{\kappa }\left(\text{z}\right)}{\partial z_{\alpha }}=-Z_{\kappa }^{-1}\int_{R^{N}}\;\kappa \left(z_{\alpha }-\theta_{\alpha }\left(\text{x}\right)\right)e^{-\beta U_{\kappa }\left(\text{x},\text{z}\right)}d\text{x}.$$

The quantity *F_k_* (***z***) in equation (5) is related to the free energy equation (1): it is a smoothed, also said mollified, version of the true free energy equation (1), which can be made as similar as desired to it by taking κ large in equation (3). How large, can be decided by observing that

(8) $$e^{-\beta F_{\kappa }\left(\text{z}\right)}=\int_{R^{n}}\;e^{-\beta F\left(\text{z}\prime \right)}e^{-\beta \frac{1}{2}\kappa \sum_{\alpha =1}^{n}\;{\left(z_{\alpha }^{\prime }-z_{\alpha }\right)}^{2}}d\text{z}\prime ,$$

i.e., *F_k_*(***z***) is a filtered version of *F*(***z***) with variance 1/*βκ*. Hence, κ should be chosen considering that $1∕\sqrt{\beta \kappa }$ sets the scale at which *F*(***z***) will be resolved. Finally, since in the same condition of large κ we also have that $-\frac{\partial F_{\kappa }\left(\text{z}\right)}{\partial z_{\alpha }}\approx -\frac{\mathrm{\partial F}\left(\text{z}\right)}{\partial z_{\alpha }}$, we obtain that the evolution equation (6) is an approximation of equation (2) [for explicit expressions of the errors this introduces, see Maragliano and Vanden-Eijnden (2006)].

The interesting property of equation (6) is that the probability distribution of the ***z*** variables obeying that dynamics is $e^{-\bar{\beta }F_{\kappa }\left(\text{z}\right)}$. This means that if we simulate the extended system equation (3) and we reconstruct the probability distribution of the ***z*** variables we can directly obtain the free energy *F_k_*(***z***). Since this holds at any artificial temperature ${\bar{\beta }}^{-1}$, we can choose it such that ${\bar{\beta }}^{-1}≳\Delta F$, where Δ*F* is an estimate of the free-energy barriers, so that the ***z*** variables will be able to cross barriers that are higher than the thermal energy at the physical temperature. Indeed, note that increasing the temperature of the CVs is equivalent to decreasing the height of the free-energy barriers.

The other important parameters to adjust to simulate equation (3) are the effective masses *μ_α_*. In practice, the condition *μ_α_* > > *m_i_* introduces an effective adiabatic separation between the ***z*** and the ***x*** variables, i.e., it guarantees that the ***x*** have time to equilibrate at the new value of ***z***. This property relies on averaging theorems for systems with multiple time scales (Papanicolaou, 1977; Vanden-Eijnden, 2003; Pavliotis and Stuart, 2008), and it yields a useful criterion to choose the effective masses *μ_α_*: specifically, the dynamics of the ***z*** must be damped so that it is possible to find a time interval over which the time average of the κ(*θ_α_*(***x***)−*z_α_)* term in equation (3) [see equation (7)] converges as if the ***z*** were essentially fixed.

TAMD shares ideas with other enhanced sampling techniques, in particular, for what concerns the extended phase-space formulation and the use of two temperatures. For the extended system, TAMD borrows from the extended Lagrangian version of metadynamics (Iannuzzi et al., 2003). It is also important to recall that a more recent version of metadynamics uses two different temperatures on the physical and collective variables (Barducci et al., 2008). As it is well-known, however, in metadynamics the CVs are pushed to exit the free-energy minima by flooding their space with Gaussian packets, which accumulate in a history-dependent term entering the equations of motion. Conversely, TAMD achieves barriers crossing by assigning a high temperature to the CVs. This simplifies the use of the technique, since in TAMD the acceleration is controlled by one parameter only (${\bar{\beta }}^{-1}$), while in metadynamics one has to specify the width, height, and dropping frequency of the Gaussians. It also makes it more efficient in principle since due to the history-dependent term the cost of a metadynamics run increases rapidly with simulation time. This is particularly true in cases where the number of collective variables is large, since filling a multi-dimensional volume requires a lot of Gaussian packets and thus increases the computational burden to calculate the history-dependent term [for possible numerical strategies to facilitate the calculation of this term, see Babin et al. (2006), Smiatek and Heuer (2011), while an extensive discussion of the parameters of metadynamics can be found in Laio et al. (2005)].

As for the use of different temperatures, efficient control of double temperature systems based on adiabaticity was introduced long ago in MD (Blöchl and Parrinello, 1992), and revised later in the context of enhanced sampling by the work of Rothlisberger [CAFES method (VandeVondele and Rothlisberger, 2002)] and Tuckerman [AFED method (Rosso et al., 2002)]. In CAFES, however, there are no additional equations of motion for the CVs: the full system is divided in a reactive subsystem (S) and the environment (E), and the dynamics of S is decoupled from E by using large masses and accelerated via a high temperature. The statistics of a CV is then extracted directly from the dynamics of S. AFED equations were also originally formulated in the space of the physical variables ***x*** only, however, collective variables are introduced in this case, and their equations of motion obtained via appropriate coordinate transformations. Using an extended system as in TAMD provides a significant simplification, especially for non-trivial ***θ***(***x***), and, in fact, the most recent version of AFED, called driven-AFED (Abrams and Tuckerman, 2008), exploits the same idea and is essentially the same as TAMD.

#### 2.1.2 Generalizations and Biophysical Applications of TAMD

As already noted, the equilibrium probability distribution of the ***z***(*t*) variables that are solution of the system equation (3) is $\rho_{\kappa }\left(\text{z}\right)\propto e^{-\bar{\beta }F_{\kappa }\left(\text{z}\right)}$, where *F_k_*(***z***) is given by equation (5). This implies that if we simulate equation (3) and we bin the values of ***z***(*t*) to get an estimate of *ρ_κ_*(***z***), we can directly reconstruct the free-energy associated to these variables (an operation called Boltzmann inversion). This was indeed the original idea for TAMD. Later (Maragliano and Vanden-Eijnden, 2008), it was demonstrated that TAMD can be used as an efficient means to rapidly browse through the relevant regions of the free-energy landscape (including those surrounded by high energy barriers), generating points where the mean forces are accurately computed at a second stage and used in a variational procedure to reconstruct the free-energy globally. This approach, termed Single-Sweep, has been successfully applied to calculate free-energy surfaces for CO (Maragliano et al., 2010), H_2_O (Lapelosa and Abrams, 2013), and O_2_ (Bucci and Abrams, 2014) diffusion in myoglobin and sarcosine oxidase.

TAMD has also been used to explore the free-energy space associated to a large number of CVs in several applications: from structure determination of solvated polymers (Lucid et al., 2012) to large-scale protein conformational transitions, such as those of the GroEL chaperonin subunit and the HIV-1 envelope glycoprotein gp120 (Abrams and Vanden-Eijnden, 2010), a maltose transporter (Vashisth and Brooks, 2012), the *β*2-adrenergic receptor (Nygaard et al., 2013), allosteric inhibition (Vashisth et al., 2013), and nucleotide release (Dror et al., 2015) in G proteins, the insulin receptor kinase (Vashisth et al., 2012a), an adenyl cyclase (Selwa et al., 2014), and the acetylcholine binding protein (Mohammad Hosseini Naveh et al., 2014).

Other attractive applications of TAMD are the determination of coarse-grained force-field parameters (Abrams and Vanden-Eijnden, 2012) and protein structure refinement from electron microscopy maps (Vashisth et al., 2012b). In particular, in the latter case, TAMD was used to enhance the flexible fitting of all-atom protein and RNA models into low-resolution density maps. In Yamamori and Kitao (2013), an interesting combination of TAMD and Replica Exchange Umbrella Sampling (REUS) was proposed. More recently, TAMD was combined with a soft-ratcheting algorithm to achieve a focused exploration of the CV space by relying on low-resolution or even qualitative experimental information (Cortes-Ciriano et al., 2015). Such an approach was successful in providing previously unavailable conformations of calmoduline-free adenyl cyclase toxin from the causative agent of whooping cough.

### 2.2 Logarithmic mean force dynamics (LogMFD)

LogMFD was introduced in Morishita et al. (2012) and further discussed in Morishita et al. (2013). In Nakamura et al. (2014), it was combined with a Density Functional Theory approach to study the conformational dynamics of a glycine peptide. Similarly to what was suggested with equation (2), in LogMFD the collective variables are considered dynamical variables, and they are evolved using the following equation of motion

(9) $$\mu_{\alpha }{\ddot{z}}_{\alpha }=-\left(\frac{\sigma \lambda }{\sigma F\left(\text{z}\right)+1}\right)\frac{\mathrm{\partial F}\left(\text{z}\right)}{\partial z_{\alpha }}+\text{ thermostat at }{\bar{\beta }}^{-1},$$

where *σ* and λ are real positive parameters discussed below, and $\bar{\beta }=1∕k_{B}\bar{T}$ is again the inverse of an effective temperature, which is in general different from the physical temperature, although in LogMFD is often set as equal to it. By comparing equations (9) with (2), we see that in LogMFD the force acting on the CVs is not simply the gradient of the free energy *F*(***z***), but instead that of its logarithmic form λ log(σ*F*(***z***) + 1). Using a logarithmic scaling for *F*(***z***) is convenient since it is a non-linear transformation, i.e., it preserves the details of the landscape at low *F*(***z***) while it smooths the regions at high values. To avoid (σ*F* + 1) < 1, *F*(***z***) is shifted by a constant *c*, i.e., *F* = *F*′ + *c*.

The advantage of using equation (9) becomes clearer if we consider the Nosé-Hoover thermostat and we introduce the conserved quantity associated to it (Nosé, 1984; Hoover, 1985),

(10) $$\hat{H}=\sum_{\alpha =1}^{n}\;\frac{1}{2}\mu_{\alpha }{\dot{z}}_{\alpha }^{2}+\lambda \text{ log}\left(\sigma F\left(\text{z}\right)+1\right)+\frac{1}{2}m_{\eta }{\dot{\eta }}^{2}+n{\bar{\beta }}^{-1},$$

where *η* is the thermostat variable and *m_η_* its fictitious mass. From equation (10), it is possible to obtain an expression for the free energy *F*(***z***),

(11) $$F\left(\text{z}\right)=\frac{1}{\sigma }\left\{\text{exp}\;\left[\frac{1}{\lambda }\left(\hat{H}\;-\;\sum_{\alpha =1}^{n}\;\frac{1}{2}\mu_{\alpha }{\dot{z}}_{\alpha }^{2}\;-\;\frac{1}{2}m_{\eta }{\dot{\eta }}^{2}\;-\;n{\bar{\beta }}^{-1}\right)\;\right]\;-1\;\right\}.$$

This indicates that we do not need to explicitly set the shifting constant *c*, but instead the conserved quantity $\hat{H}$ needs to be set at the beginning of a LogMFD run to guarantee *F*(= *F*′ + *c*) ≥ 0. In particular, $\hat{H}$ should be chosen to satisfy

(12) $$\left(\hat{H}-\sum_{\alpha =1}^{n}\;\frac{1}{2}\mu_{\alpha }{\dot{z}}_{\alpha }^{2}-\frac{1}{2}m_{\eta }{\dot{\eta }}^{2}-n{\bar{\beta }}^{-1}\right)\equiv F^{*}\left(\text{z}\right)\geq 0.$$

Since the average of the kinetic energy of ***z*** and the terms associated with the thermostat variables can be easily determined for a defined temperature ${\bar{\beta }}^{-1}$, one can roughly estimate an appropriate value of $\hat{H}$. In order to take full advantage of the logarithmic form, F*/*λ* should be close to 0 at the bottom of the free-energy landscape. Obviously, we do not know the exact position of the minimum of *F*(***z***) before running a LogMFD calculation, but since results are not sensitive to the choice of $\hat{H}$, only a rough estimate of the conserved quantity is sufficient.

Equation (11) shows that a free-energy profile *F*(***z***) can be reconstructed directly as the ***z***(*t*) evolve in a LogMFD run obtained by solving equation (9). The key to this direct estimation is the existence of the conserved quantity $\hat{H}$, and hence, in equation (9), it is possible to use any Nosé-Hoover (NH) type thermostat [NH chains (Martyna et al., 1992), recursive NH (RNH) (Morishita, 2010), or Gaussian moment thermostats (Liu and Tuckerman, 2000)], as long as it exists a conserved quantity analogous to $\hat{H}$ in equation (10). Similarly to equation (2), the mean force in equation (9) can be estimated via conditional averages using the blue moon ensemble or with harmonic restraints as in equation (7).

Moreover, to avoid the problems with averaging and re-initialization discussed in Section 2.1.1, a concurrent evolution scheme for ***z***(*t*) and ***x***(*t*) as in TAMD system of equations equation (3) can be envisioned, where the ***z***(*t*) are damped with respect to the ***x***(*t*) by larger masses.

Two important parameters are introduced in LogMFD, *σ* and *λ*. For simplicity, *λ* is usually set as 1/*σ*. The role of *σ* is to flatten the free-energy landscape during the evolution of ***z***(*t*). The choice of optimal σ comes from a trade-off between a large value which would enhance barrier crossing and a small value to reduce numerical errors originating from the exponential form of *F*(***z***) equation (11). A reasonable choice is to set *σ* such that ${\bar{\beta }}^{-1}$ is of the order of log(*σ*Δ*F* + 1)/*σ*, where Δ*F* is an estimate of the free-energy barrier and we have used *λ* = 1/*σ*. Note that, as a consequence, the temperature ${\bar{\beta }}^{-1}$ in LogMFD is usually lower than then the one needed in TAMD for barrier crossing (${\bar{\beta }}^{-1}\approx \Delta F$), and it can be even lower than the physical temperature. However, this comes at the price of setting two more parameters, *σ* and *λ*. For an extensive investigation on the role of the different LogMFD parameters, we refer the reader to Morishita et al. (2013).

### 2.3 Multiscale enhanced sampling (MSES)

The MSES technique was introduced in Moritsugu et al. (2010) and applied so far to study the disorder to order transition in a sortase protein (Moritsugu et al., 2012), and the formation of a Barnase–Barstar complex (Moritsugu et al., 2014a). In MSES, a physical system ***x*** is again coupled to a set of extra variables ***z*** via the use of CVs functions ***θ***(***x***). The equations of motion for MSES can be written as

(13) $$\left\{\begin{array}{c} m_{i}{\ddot{x}}_{i}\;=-\frac{\mathrm{\partial V}\left(\text{x}\right)}{\partial x_{i}}\;-\kappa \;\sum_{\alpha =1}^{n}\;\;\;\left(\theta_{\alpha }\left(\text{x}\right)\;-\;z_{\alpha }\right)\frac{\partial \theta_{\alpha }\left(\text{x}\right)}{\partial x_{i}}\;+\;\text{thermostat}\;\text{at}\;\beta^{-1},\;\\ \mu_{\alpha }{\ddot{z}}_{\alpha }=\kappa \left(\theta_{\alpha }\left(\text{x}\right)-z_{\alpha }\right)-\frac{\partial V^{\mathrm{CV}}\left(\text{z}\right)}{\partial z_{\alpha }}+\text{thermostat}\;\text{at}\;{\bar{\beta }}^{-1}.\; \end{array}\right.$$

At variance with TAMD equations (3), the system equation (13) contains also a force on the ***z*** variables coming from a potential energy acting on these variables, *V^CV^* (***z***) [a version of MSES using multiple and mutually interacting ***z*** sets was presented in Moritsugu et al. (2014b)]. This potential is arbitrarily chosen based on prior knowledge or experimental information. In general, it is designed to guide sampling by relying on well-established coarse-grained models. For example, in the study of the Barnase–Barstar complex (Moritsugu et al., 2014a), C*α*–C*α* distances were used as CVs, and the CV potential *V^CV^* (***z***) was prepared as the sum of two terms representing intra- and inter-molecular interactions. For intra-molecular interactions the potential function of the C*α* elastic network model was used (Tirion, 1996), while for inter-molecular interactions the Lennard–Jones potential was applied to selected C*α* atom pairs.

Another difference with TAMD is that in MSES the adiabatic separation between ***x*** and ***z*** variables is not necessarily assumed, although in practice the values of *μ_α_* and ${\bar{\beta }}^{-1}$ can be set to achieve maximal sampling efficiency. Rather, it is suggested to use equation (13) in a Hamiltonian replica exchange scheme (Fukunishi et al., 2002), by introducing a set of replicas of the extended (***x***, ***z***) system, each characterized by a different value of the coupling constant κ. Harvesting data from the replica with κ = 0 enable the elimination of the biasing coupling potential, recovering a canonical probability distribution for the ***x*** and thus the possibility to reconstruct free-energy surfaces from any other CV by simply monitoring their probability distribution. In practice, 20 and 12 replicas were used to study the sortase disorder to order transition and the Barnstar–Barnase complex, respectively. Note that in principle any kind of biasing potential can be used. However, using stronger bias will lead to a larger number of replicas for unbiasing to κ = 0.

## 3 Methods to Determine Reactive Paths

In many cases, rather than reconstructing large portions of the free-energy space, one is interested in directly finding paths connecting different minima in this landscape. This different perspective was first introduced by Pratt (1986) using a “chain-of-states" linking two minima, and successively developed by Chandler and collaborators in the transition path sampling (TPS) technique (Bolhuis et al., 2002), where the basic idea is to reconstruct the ensemble of transition paths directly from pieces of dynamical trajectories joining the two minima. These pioneer methods and many others such as those of Elber and collaborators [see Májek et al. (2008) for a comprehensive review], the Nudged Elastic Band (Jonsson et al., 1998) or the zero temperature string method (E et al., 2002, 2007b), were developed to work in the full Cartesian space, thus searching for transition paths in the potential energy space. This approach becomes difficult when the dimensionality of the system is large (as it is often encountered in relevant biophysical problems), since in this case the potential energy space is very noisy (i.e., rugged on a scale smaller than the thermal energy), and as a consequence the reactive paths are highly twisted. To get around this hurdle, the string method in collective variables algorithm was introduced in Maragliano et al. (2006) to find reaction paths in free-energy space, by identifying them as Minimum Free Energy Paths (MFEPs). The main advantage of this approach with respect to those working in potential energy space is that the free energy is usually much smoother. Moreover, with respect to techniques for sampling and reconstructing globally the free-energy space, the string method permits the use of a larger number of collective variables.

The basic idea of the string method is to represent the transition path between two free-energy minima in the space of a set of CVs ***θ***(***x***) = (*θ_1_*(***x***), … ,*θ_n_*(***x***)) as a curve (the string), discretized in a collection of points called images. The positions of these images are evolved until convergence using information harvested from MD simulations of replicas of the full system, constrained or restrained at the CV values that define the images. To avoid that all images collapse in the two minima, a curve parametrization is enforced during the evolution. The path obtained at convergence depends on the version of the algorithm used. The original version in Maragliano et al. (2006) converges to the MFEP, while other successive versions yield a slightly different path (more details will be given below). A detailed comparison of different string method algorithms and of the paths they converge to is presented in Maragliano et al. (2014) [for a comprehensive review, see also E and Vanden-Eijnden (2010)]. In the next paragraph, we briefly review the definition of these paths and their relevance in the study of reactive events, while in Section 3.1.2, we describe the main features of the on-the-fly version of the string method in CVs.

### 3.1 The on-the-fly string method in collective variables

#### 3.1.1 The Minimum Free-Energy Path and its Relevance

Given two minima *A* and *B* of the free energy *F*(***z***), the MFEP was defined in Maragliano et al. (2006) as the curve that connects *A* and *B* and to which the vector *M*(***z***) ⋅ ▽*F*(***z***) is everywhere tangent. Hence, it is the curve satisfying the equation

(14) $${\left[M\left(\text{z}\right)\cdot \nabla F\left(\text{z}\right)\right]}^{⊥}=0$$

where the superscript ⊥ indicates projection in the direction perpendicular to the curve itself. In equation (14), ▽*F*(***z***) is the gradient of the free energy (the mean force), and *M*(***z***) is a metric tensor which accounts for the curvilinear nature of the CVs, defined component-wise by the conditional average

(15) $$M_{\alpha \beta }\left(\text{z}\right)={\left\langle \sum_{i=1}^{N}\;\frac{1}{m_{i}}\;\frac{\partial \theta_{\alpha }\left(\text{x}\right)}{\partial x_{i}}\frac{\partial \theta_{\beta }\left(\text{x}\right)}{\partial x_{i}}\right\rangle }_{\theta \left(\text{x}\right)=\text{z}}$$

or explicitly

(16) $$\begin{array}{l} M_{\alpha \beta }\left(\text{z}\right)=e^{F\left(\text{z}\right)∕k_{B}T}Z^{-1}\int d\text{x}\sum_{i=1}^{N}\frac{1}{m_{i}}\frac{\partial \theta_{\alpha }\left(\text{x}\right)}{\partial x_{i}}\frac{\partial \theta_{\beta }\left(\text{x}\right)}{\partial x_{i}}e^{-V\left(\text{x}\right)∕k_{B}T}\delta \left(\theta \left(\text{x}\right)-\text{z}\right). \end{array}$$

As already anticipated in Sections 1 and 2.1.1, the mean force can be obtained using the *blue moon* ensemble technique (Ciccotti et al., 2005) or by harmonic restraints [see equation (7)]. The same is valid for the tensor *M*(***z***).

The MFEP equation (14) is the curve obtained by the original string method algorithm in CVs described in Maragliano et al. (2006). The importance of the MFEP in order to understand the mechanism of the transitions between a reactant state *A* and the product state *B* is established within the framework of Transition Path Theory (Vanden-Eijnden, 2007; E and Vanden-Eijnden, 2010). Indeed, using TPT, it is possible to demonstrate that in appropriate regimes, and when the reaction is described in the space of a set of collective variables, the MFEP is the most probable transition path between *A* and *B*.

Let us recall here briefly this result, while for more details we refer any interested reader to Vanden-Eijnden (2007) and E and Vanden-Eijnden (2010). From TPT, the key quantity to describe the statistical properties of the reactive trajectories is the committor *q*(***x***, ***v***), a function of the positions and velocities of the physical system, which gives the probability that the trajectory initiated at (***x***, ***v***) will reach the product *B* before the reactant *A*. Via the committor function it is possible to obtain analytic expressions for the probability density of reactive trajectories, their probability current, and the rate of the reaction, and for this reason, it is the *optimal* reaction coordinate. The committor satisfies a Fokker–Planck equation, which is too complicated to be solved by standard numerical methods when the dimensionality of the system is large. To get around this difficulty, in the string method it is assumed that *q*(***x***, ***v***) can be approximated by a function that depends only on the positions ***x***, via a set of collective variables ***θ***(***x***) = (*θ_1_*(***x***), … , *θ_n_*(***x***)), i.e., *q*(***x***, ***v***) ≈ *Q*(***θ***(***x***)).

As shown in Maragliano et al. (2006), *Q*(***z***) satisfies a much simpler Fokker–Planck equation, the equation for the committor function associated to the following overdamped equation for the collective variables (α = 1, … ,*n*)

(17) $$\begin{array}{l} \gamma_{z}\frac{dz_{\alpha }}{\mathrm{dt}}=\sum_{\beta =1}^{n}\;\left(-M_{\alpha \beta }\left(\text{z}\right)\frac{\mathrm{\partial F}\left(\text{z}\right)}{\partial z_{\beta }}+k_{B}T\frac{\partial }{\partial z_{\beta }}M_{\alpha \beta }\left(\text{z}\right)\right)+\sqrt{2k_{B}T}\sum_{\beta }\;M_{\alpha \beta }^{1∕2}\left(\text{z}\right)\eta_{\beta } \end{array}$$

where *M_αβ_* is the tensor defined in equation (16) and *γ_z_* is an artificial friction coefficient. Notably, this reasoning does not require to specify a value for *γ_z_*. The discussion above implies that we can use equation (17) to study the mechanism of the reaction from *A* to *B*. Indeed, if the temperature is low compared to the relevant free-energy barriers, the trajectories solution of equation (17) concentrate around a curve in collective variable space which (i) connects the two minima *A* and *B* of the free-energy surface via a saddle point and (ii) is everywhere tangent to the vector field *M*(***z***)▽*F*(***z***) [consider the limit *T* → 0 of equation (17)]. In other words, the reactive trajectories in CV space lie along the MFEP defined as in equation (14). Hence, the string method is a practical procedure to identify globally these transition paths by evolving their representative curve in collective variable space.

It is also possible to include in the definition of the reaction path equation (14) the extra term in the drift from equation (17), which contains the divergence of *M*(***z***), *k_B_T*Σ*_β_*∂*M_α,β_/∂z_β_*. In this case, the relevant path becomes the curve solution of

(18) $$0={\left[-M\left(\text{z}\right)\nabla F\left(\text{z}\right)+k_{B}T\;\nabla \cdot M\left(\text{z}\right)\right]}^{⊥}.$$

where ▽ ⋅ *M*(***z***) is compact notation for the divergence of *M*(***z***). The path solution of equation (18) is the one identified by a particular implementation of the on-the-fly string method (Maragliano and Vanden-Eijnden, 2007) (see below), and also by other successive variants of the method (Pan et al., 2008; Johnson and Hummer, 2012). Note that the solution of equation (18) does not go through the same critical points as the MFEP, i.e., unlike the MFEP it cannot be used to identify the saddle points on the free-energy landscape. However, because the extra term in equation (18) is proportional to *k_B_T* it should not have much influence, and the differences between the MFEP solution of equation (14) and the curve solution of equation (18) are expected to be small and difficult to distinguish from numerical fluctuations. This was indeed the result observed for the specific cases of solvated (Maragliano and Vanden-Eijnden, 2007) and gas-phase (Maragliano et al., 2014) alanine dipeptide, using dihedral angles as collective variables.

#### 3.1.2 The on-the-Fly String Method Algorithm

In the original formulation of the string method in CVs (Maragliano et al., 2006), one MD replica per image is used, and the force driving the evolution of the images is the product of the mean force (the gradient of free energy) and the tensor *M*(***z***), both of which can be expressed in terms of expectations conditional on ***θ***(***x***) = ***z***. This procedure requires that once the new values of the images are obtained, the MD system is re-initialized to have that at each image the ***θ***(***x***) are close to the new ***z***.

Successively (Maragliano and Vanden-Eijnden, 2007), a different version of the string method, called on-the-fly, was developed, which exploits the same ideas at the basis of TAMD that were described in Section 2.1.1. The basic idea of this other algorithm is to evolve the string images concurrently with the attached replicas of the MD system, which provide on-the-fly the data needed to the evolution of the images. This is attained by introducing an extended system comprising the physical and collective variables, which are evolved concurrently. Similarly to TAMD, the motion of the string images is dampened with respect to the MD replicas, so that, by relying on the same averaging theorems for systems with multiple time scales that are at the basis of TAMD, one can bypass the computation of the string driving force via averaging. This approach also eliminates the need for re-initializing the MD systems after every string update, thus providing a simpler, more stable and more efficient algorithm.

There are two versions of the on-the-fly string method algorithm, which differ by the number of independent MD replicas attached to each image. Indeed, it was shown in Maragliano and Vanden-Eijnden (2007) that when two replicas per image are used, the algorithm converges to the MFEP equation (14), while when only one replica is used the algorithm converges to the curve defined by equation (18). We illustrate here for simplicity the one-replica version, and we address the reader to Maragliano and Vanden-Eijnden (2007) for more details about the one with two replicas and the method in general.

It is useful to present the method in the continuous formulation, i.e., by considering the moving string as a time-dependent curve parametrized as {***z***(*s, t*) : *s* ∈ [0, 1]}. Such curve is evolved concurrently with a one-parameter family of replicas of the MD system {***x***(*s, t*) : *s* ∈ [0, 1]}. Written component-wise for α = 1, … , *n* and *i* = 1, … , *N*, the equations for the concurrent dynamics are

(19) $$\left\{ \begin{array}{l} \gamma_{z}{\dot{z}}_{\alpha }(s,t)=\sum_{\beta =1}^{n}{\tilde{M}}_{\alpha \beta }(x(s,\;t))\;\kappa (\theta_{\beta }(x(s,\;t)-z_{\beta }(s,\;t)))+\Lambda (s,\;t)z^{\prime }\;\alpha \;(s,\;t)\\ m_{i}\;{\ddot{x}}_{i}\;(s,\;t)=-\frac{\partial V(x(s,\;t))}{\partial x_{i}}-\kappa \sum_{\beta =1}^{n}\left(\theta_{\beta }(x(s,\;t\right)-z_{\beta }(s,\;t)))\frac{\partial \theta_{\beta }(x(s,\;t))}{\partial x_{i}}\\ \;+\text{thermostat at }\beta^{-1} \end{array}\right.$$

where γ*_z_* is an effective friction term acting on the collective variables and

(20) $${\tilde{M}}_{\alpha \beta }\left(\text{x}\right)=\sum_{i=1}^{N}\;\frac{1}{m_{i}}\;\frac{\partial \theta_{\alpha }\left(\text{x}\right)}{\partial x_{i}}\frac{\partial \theta_{\beta }\left(\text{x}\right)}{\partial x_{i}}.$$

In equation (19), Λ(*s, t*)*z*′α(*s*, *t*), with *z′*_α_ = ∂*z*_α_/∂*s*, is a Lagrange multiplier term used to enforce the particular parametrization chosen for the curve (for example, normalized arc length). Note that, at variance with equation (3), in equation (19), we use first order dynamics for the ***z*** variables. This is done for simplicity and because the evolution of ***z***(*s, t*) in equation (19) has a different purpose than that of ***z***(*t*) in equation (3), i.e., it is not an exploratory dynamics but rather a steepest descent along the driving force. Analogously to equation (3), however, the system equation (19) describes the evolution of the extended system (***x***, ***z***) under the effect of the potential energy equation (4). Here, the friction coefficient γ*_z_* plays the role of the masses μ_α_ in equation (3).

Following the same reasoning illustrated in Section 2.1.1, in Maragliano and Vanden-Eijnden (2007), it was shown that by simulating the system equation (19) with κ and γ*_z_* sufficiently large, the ***z***(*s, t*) evolve according to an effective equation obtained by averaging the equations for ***z***(*s, t*) in equation (19) with respect to the probability density function for the equation for ***x***(*s, t*) in equation (19) at ***z***(*s, t*) = ***z***(*s*) fixed. In this case, the effective equation is

(21) $$\begin{array}{l} \gamma_{z}{\dot{z}}_{\alpha }\left(s,t\right)=\sum_{\beta =1}^{n}\;\left(-M_{\alpha \beta }\left(\text{z}\left(s,t\right)\right)\frac{\mathrm{\partial F}\left(\text{z}\left(s,t\right)\right)}{\partial z_{\beta }}\right.\left.+k_{B}T\frac{\partial }{\partial z_{\beta }}M_{\alpha \beta }(\text{z}\left(s,t\right)\right)+\lambda \left(s,t\right){z\prime }_{\alpha }\left(s,t\right). \end{array}$$

It can be easily seen that the steady state solution of equation (21) is precisely the curve equation (18). Hence, if we evolve a string {***z***(*s*) : *s* ∈ [0, 1]} by simulating equation (19) using large but finite values of κ and γ*_z_*, this will converge to the curve defined by equation (18).

As a final comment, note that, at variance with equation (3), the system equation (19) does not include a thermostat acting on the ***z***(*s, t*) variables. This generates a steepest descent dynamics for ***z***(*s, t*) on the free-energy surface, which is appropriate when the surface is smooth. In cases where the free-energy landscape is rugged, albeit on a scale smaller than the barrier defining the transition state, a thermostat term can be added to the ***z***(*s, t*) equation in order to let the string overcome local free-energy asperities. This idea was explored in Vanden-Eijnden and Venturoli (2009) and Stober and Abrams (2012). However, it is important to note that in practical implementations of the system equation (19) the timescale separation between ***x*** and ***z*** is usually approximate, which means that ***z***(*s, t*) satisfy the limiting equation (21) only up to a residual error. This error produces a noise on the evolution of ***z***(*s, t*), which is comparable to a low-temperature thermostat. Basing on our experience, we believe that it is possible to impose a separation of timescales such that ***z***(*s, t*) obey equation (21) to a satisfactory accuracy, but with enough residual noise to navigate over the local ruggedness of *F*(***z***).

#### 3.1.3 Biophysical Applications of the OTF String Method

So far, the OTF string method has been successfully utilized to study the ligand-induced conformational change of adenylate kinase (Matsunaga et al., 2012), the formation of a stalk between two apposed membrane bilayers (Müller et al., 2012), and the normal-to-amyloidogenic isomerization of β2-microglobulin (Stober and Abrams, 2012). Notably, in Stober and Abrams (2012), the authors introduce a clever implementation of the method by simulating a single MD system comprising all the string images embedded in a single, large solvent box. In Ovchinnikov et al. (2011), an intermediate variant between the original and on-the-fly string method was employed to study the conformational transition of myosin VI. Finally, in Zinovjev et al. (2013) the on-the-fly string method was used in combination with a Quantum Mechanics/Molecular Mechanics approach to study the reaction catalyzed by guanidinoacetate methyltransferase.

### 3.2 The onsager–machlup multiscale enhanced sampling method

An enticing idea to sample transition paths has been developed in Fujisaki et al. (2013) by combining the MSES technique presented in Section 2.3 with an Onsager–Machlup (OM) action approach (MSES-OM). In MSES-OM, an extended system is again considered, comprising the physical and CV variables (***x***, ***z***), and a global OM action is defined as the sum of the OM actions for the ***x*** and ***z*** variables and an interaction term, S(***x***, ***z***) = *S*(***x***) + *S^CV^*(***z***) + κ*S*^int^(***x***, ***z***). The basic idea is that the CG variables ***z*** move rather freely in path space, dragging with them the ***x*** variables. Similarly to MSES, a replica exchange framework is introduced by using many replicas of the system (***x***, ***z***), each one with a different values of *k*. The canonical OM path weight exp(−*S*[(***x***)]) is then recovered from the replica with κ = 0. The MSES-OM method has been extensively investigated in Fujisaki et al. (2013) by applying it to study the unlooping kinetics of a model polymer, revealing a substantial gain in computational time with respect to conventional OM method.

The advantages in using action methods are that they provide dynamical information on the transition studied, and from a numerical standpoint that the time step for the action discretization can be taken much larger than the typical MD step (≈ 1fs). On the other hand, known limitations of the approach are that one is required to know beforehand the total time of the transition, and numerical difficulties due to the use of high order derivatives of the potential (Vanden-Eijnden and Heymann, 2008).

## 4 Concluding Remarks

We have reviewed a few enhanced sampling methods that allow to accelerate MD simulations and to study rare reactive events. The common feature of these methods is that they rely on extending the phase space of the physical system under study by adding a set of extra variables also considered as dynamical ones, linked to the original system via CV functions of the physical coordinates. This setting allows efficient exploration of the CV space, i.e., of the free-energy landscape of the original system with respect to the CVs. The various methods achieve this exploration via different means. In TAMD, the CVs are evolved at a higher temperature than the physical variables, so that they can efficiently cross free-energy barriers higher than the physiological temperature. In LogMFD, a logarithmic transformation of the free energy is employed together with the conserved quantity from deterministic dynamics to reconstruct directly the free energy during CV evolution. In MSES, a model potential is introduced for the CVs also, and different replicas of the extended system are used in a replica exchange framework, each with a different value of the coupling parameter between the CVs and the original system. The statistical properties of the unbiased physical system are then reconstructed from the replica with zero coupling. We have also reviewed path sampling methods based on the same extended phase-space ideas, namely as the on-the-fly string method and the Onsager–Machlup action MSES.

## Conflict of Interest Statement

The authors declare that the research was conducted in the absence of any commercial or financial relationships that could be construed as a potential conflict of interest.
